# Involvement of Sodium Nitroprusside (SNP) in the Mechanism That Delays Stem Bending of Different Gerbera Cultivars

**DOI:** 10.3389/fpls.2017.02045

**Published:** 2017-11-28

**Authors:** Aung H. Naing, Kyoungsun Lee, Kyoung-Ook Kim, Trinh N. Ai, Chang K. Kim

**Affiliations:** ^1^Department of Horticultural Science, Kyungpook National University, Daegu, South Korea; ^2^School of Agriculture and Aquaculture, Tra Vinh University, Tra Vinh, Vietnam

**Keywords:** antioxidant activity, bacterial density, genotype, lignin, xylem blockage

## Abstract

Longevity of cut flowers of many gerbera cultivars (*Gerbera jamesonii*) is typically short because of stem bending; hence, stem bending that occurs during the early vase life period is a major problem in gerbera. Here, we investigated the effects of sodium nitroprusside (SNP) on the delay of stem bending in the gerbera cultivars, Alliance, Rosalin, and Bintang, by examining relative fresh weight, bacterial density in the vase solution, transcriptional analysis of a lignin biosynthesis gene, antioxidant activity, and xylem blockage. All three gerbera cultivars responded to SNP by delaying stem bending, compared to the controls; however, the responses were dose- and cultivar-dependent. Among the treatments, SNP at 20 mg L^-1^ was the best to delay stem bending in Alliance, while dosages of 10 and 5 mg L^-1^ were the best for Rosalin and Bintang, respectively. However, stem bending in Alliance and Rosalin was faster than in Bintang, indicating a discrepancy influenced by genotype. According to our analysis of the role of SNP in the delay of stem bending, the results revealed that SNP treatment inhibited bacterial growth and xylem blockage, enhanced expression levels of a lignin biosynthesis gene, and maintained antioxidant activities. Therefore, it is suggested that the cause of stem bending is associated with the above-mentioned parameters and SNP is involved in the mechanism that delays stem bending in the different gerbera cultivars.

## Introduction

Due to its diverse flower colors and shapes, gerbera is one of the 10 most popular commercial cut flowers in the world (Flower Council of Holland, 2007). However, the vase life of cut gerbera flowers is often short due to stem bending, which precedes wilting of the ray petals. Generally, stem bending might relate to the lack of mechanical support, particularly in the xylem (Steinitz, 1983; Marousky, 1986), and the lack of sclerenchyma cells in the stem below the floral head (Dubuc-Lebreux and Vieth, 1985; Perik et al., 2012), which contains high levels of lignin. In addition, stem bending can also occur due to xylem blockage by bacteria, which results in low water uptake and loss of sufficient turgor to maintain the weight of the flower head (van Doorn and de Witte, 1994). Moreover, phenylalanine ammonia lyase (PAL) activity is positively associated with stem turgidity in gerbera (Mencarelli et al., 1995; Ferrante et al., 2007; Ferrante and Serra, 2009) because it catalyzes the deamination of phenylalanine to *trans-*cinnamic acid, which is used to synthesize many phenolic and lignin-like compounds associated with plant metabolism (Brown, 1990). In addition, the role of PAL in the induction of phenol accumulation leading to tissue lignification has been observed in several plants (Iriti and Faoro, 2003; Liu et al., 2005). However, gerberas are not sensitive to ethylene (Reid, 1989); in addition, treatment with ethylene inhibitors also did not prevent stem bending. Therefore, ethylene did not appear to be directly involved in the vase life of gerbera flowers because it does not address problems such as lack of lignin, PAL, and xylem blockage. Moreover, most previous studies have not found any link between ethylene production and stem bending in gerbera flowers (Nair et al., 2003; Meman and Dabhi, 2006; Prashanth and Chandrasekhar, 2007; Liu et al., 2009; Solgi et al., 2009), while the link between bacterial blockage and stem bending was mainly discussed.

To overcome the problems that cause stem bending of cut gerbera flowers, various antimicrobial agents, such as silver nanoparticles (Liu et al., 2009; Solgi et al., 2009), silver nitrate, 8-hydroxyquinoline citrate (Nair et al., 2003; Meman and Dabhi, 2006; Prashanth and Chandrasekhar, 2007), and essential oils, such as carvacrol and thymol (Solgi et al., 2009), have been tested. The antimicrobial agents extended the vase life of cut gerbera flowers by strongly reducing bacterial counts in vase water and in stem vessels, in which bacteria can cause bacterial xylem blockage, thereby improving water uptake and maintaining greater relative fresh weight (RFW).

Despite the application of various antimicrobial agents to extend the vase life of gerbera, the role of sodium nitroprusside (SNP), a nitric oxide donor that is involved in multiple modes of action associated with vase life, has not been used to date for this flower. Indeed, SNP has improved the vase life of various cut flowers, such as rose, gladiolus, and carnation (Zeng et al., 2011; Liao et al., 2013; Dwivedi et al., 2016; Naing et al., 2017), by downregulating ethylene biosynthesis and senescence-associated genes and by enhancing antioxidant activity. In addition, SNP has shown antibacterial activity by inhibiting the growth of *Clostridium sporogenes, Staphylococcus aureus*, and *Escherichia coli*, which are the most commonly observed bacteria in the xylem of cut flowers (Joannou et al., 1998; Seabra et al., 2010; Martínez-Ruiz et al., 2011; Cardozo et al., 2014). Nitric oxide also positively affected lignification (Böhm et al., 2010).

Due to the involvement of nitric oxide in the inhibition of bacterial growth and in the lignification pathway, in addition to other mechanisms associated with vase life, stem bending mainly caused by bacterial xylem blockage and low lignin content could be addressed by using SNP as a nitric oxide donor. However, role of SNP in activation of PAL associated with stem rigidity has not been reported so far. Hence, it is interesting to study how SNP relates to the above-mentioned parameters involved in stem bending.

Therefore, we investigated the role of SNP in stem bending by measuring various parameters, including bacterial xylem blockage, RFW, expression of a lignin biosynthesis gene and the *PAL* gene, which is a precursor of lignin, and variation in antioxidant activity, during floral vase life.

## Materials and Methods

### Plant Material

**FIGURE 1:**
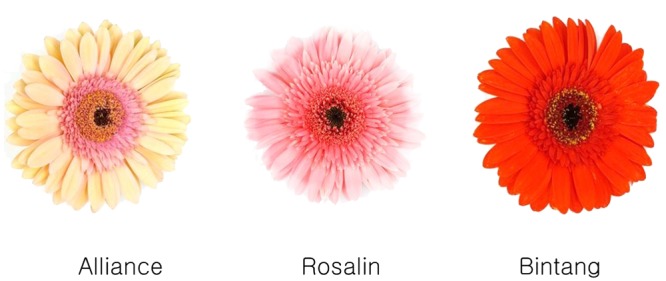
Showing flower colors of the different gerbera cultivars (Alliance, Rosalin, and Bintang) used in this study.

Three gerbera (*Gerbera jamesonii*) cultivars (Alliance, Rosalin, and Bintang) were released from their originator (Florist Holland B.V.) and have become very popular (**Figure 1**); however, their reduced vase life is a challenge for flower users. Hence, we chose these cultivars as subjects of this experiment. The cultivars were grown in fine soil (silty clay loam type) beds that were well-drained and plants were raised in a greenhouse from January to May 2016. Recommended nutrient solutions were applied as necessary following the cultivator’s instructions (Rural Development Administration [RDA], South Korea). The greenhouse was maintained with favorable environmental factors, including temperature (at 17 to 28°C during the day and 14 to 18°C at night), light (14 h light photoperiod with the help of supplemental lighting, at approximately 45.36 molm^-2^ d^-1^), and relative humidity (70%), for the production of quality cut flowers. The flowers grown in the greenhouse that were used for this experiment were 30 km from the laboratory and were graded to be of marketable quality. Once they arrived at the laboratory, the flowers were placed in distilled water and their stems were re-cut to a length of approximately 40 cm, in accordance with the marketable size.

### Treatment with SNP

Stock solutions of SNP (Enzo Life Sciences) were prepared following the manufacturer’s instructions. Seven flower stems, which had approximately the same fresh weight, were placed into 1-L plastic bottles (vases) filled with 600 mL distilled water containing different concentrations of SNP [0 (control), 1, 5, 10, 15, or 20 mg L^-1^] for 24 h. To avoid photodegradation of SNP (release of a nitrosyl ligand and a cyanide ion), the vases were wrapped with aluminum foil during the SNP treatment. Next, the treated stems were thoroughly washed under tap water and replaced into the vases containing 600 mL distilled water. The vases were then maintained in a growth chamber with a light intensity of 20 μmol^-2^ m^-2^ s^-1^ for 12 h, at 23°C and 60–70% relative humidity. There were three vases (21 flowers) per treatment and the experiment was repeated three times.

The initial fresh weight of all flowers in this experiment was recorded, after which five flowers among them were separated to evaluate RFW and vase life throughout the experiment. The remaining flowers were used for transcriptional analysis of a lignin biosynthesis and PAL genes, the investigation of xylem blockage, and antioxidant activity measurements.

### Vase Life and Relative Fresh Weight (RFW)

The vase life of each flower was determined when flower stems showed bending. From the control stems and those treated with SNP optimal concentrations (20 mg L^-1^ for Alliance, 10 mg L^-1^ for Rosalin, and 5 mg L^-1^ for Bintang), the fresh weight was measured every 3 days and the RFW was calculated using the formula: RFW (%) = (FWt/FW0) × 100, where FWt is the fresh weight of a stem (g) at days 3, 6, or 9 and FW0 is the initial fresh weight of the stem (g) at day 1 (He et al., 2006).

### Determination of Bacterial Density in the Vase Solution

The bacterial densities in vase solutions of the control and SNP treatments were determined on day 9 when most of the control flower stems exhibited stem bending. Briefly, 1 mL of vase solution was taken from the control and SNP treatment vase solutions and the optical density (OD_600_) was measured using a spectrophotometer. Five samples per treatment were used and the analysis was repeated three times.

### RNA Extraction and Quantitative Real Time PCR (qRT-PCR) Analysis

Total RNA was extracted from 100 mg of stem segments cut about 10 cm below the flower heads (at the bending location) from the treatments that were observed to give the longest vase life (20 mg L^-1^ for Alliance, 10 mg L^-1^ for Rosalin, and 5 mg L^-1^ for Bintang), along with the respective controls, using the RNeasy Plant Mini Kit (Qiagen, Hilden, Germany). The cDNA was synthesized from 1 μg of total RNA using an oligo dT_20_ primer and a reverse transcription kit (ReverTra Ace-á, Toyobo, Japan). Then, transcript levels of the lignin biosynthesis gene (*GCCoAOMT-3*) and *PAL* gene, a precursor of the lignin and flavonoid biosynthetic pathways, were measured using the StepOnePlus Real-Time PCR system (Thermo Fisher Scientific, Waltham, MA, United States) (Ai et al., 2016). To confirm the amount of template RNA, fragments of gerbera *2-pyrone synthase (2PS)* were used as the internal control. The primers and PCR conditions for the detected genes are listed in **Table 1**. Five samples per treatment (five replicates) were used and the analysis was performed at three different time points (days 3, 6, and 9).

**Table 1:** Primer sequences used for detection of genes related to lignin biosynthesis and PAL activity by qRT-PCR.

Gene	Primer sequence (5′–3′)	PCR condition
*GCCoAOMT-3*	F- CCT GCT TTG CCC GTT CTT G R- CAG AGC CGT TCC ATA GGG TG	95°C (10 min) → [95°C (15 s) → 57°C (1 min) → 72°C (35 s)] × 40 cycles → 95°C (15 s) → 59.3°C (1 min) → 95°C (15 s)
*PAL*	F- TACACGGTGGCAACTTCCAA R- ACTAGGGTTTTGACCGCCAG	
*2-pyrone synthase (2PS)*	F- CAA AGA AGC CGC AGT CAA GG R- AGC GTT TGA CTG AAG GGG AG	

### Determination of Antioxidant Activity

Stems were cut from controls and SNP treatments of each cultivar at three time points (days 3, 6, and 9) and were frozen for the analysis of antioxidant activity. For measurements of 2,2-diphenyl-1-picrylhydrazyl (DPPH) and 2,2′-azino-*bis*(3-ethylbenzothiazoline-6-sulfonic acid) (ABTS), 5 g of frozen stem segments were used and the analyses were performed following the methods of Kim et al. (2014). Five samples per treatment were used and the analysis was repeated three times.

### Scanning Electron Microscopy

To investigate the xylem status of stems in all cultivars, stem segments 3 mm long were excised from the base of control stems and SNP-treated stems on days 0, 3, and 9. The stem segments were then fixed in a formalin-acetic acid-alcohol (FAA) solution and kept overnight according to the protocol of Naing et al. (2015). The samples were then dehydrated for 10 min using an ethanol concentration series (25, 50, 70, 85, and 100%). The dehydrated samples were then dried to the critical point at room temperature, coated with gold-palladium on a Quick Cool Coater (Sanyu-Denshi, Japan), examined under a scanning electron microscope (JEOL, Ltd., Tokyo, Japan), and photographed.

### Statistical Analysis

This experiment was conducted using a completely randomized design (CRD). Data were analyzed using analysis of variance (ANOVA) in SPSS version 11.09 (IBM Corporation, Armonk, NY, United States) and are presented as the mean ± standard deviation (SD) of three replicates. Significant differences among the means were declared at *p* < 0.05 or 0.01.

## Results

### Vase Life and RFW

**FIGURE 2:**
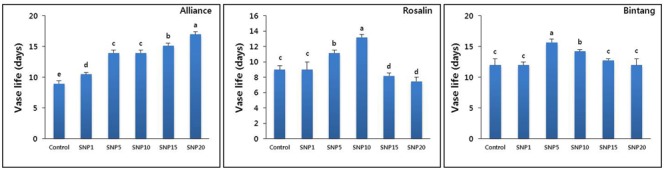
The effect of different concentrations of SNP on vase life of different gerbera cut flowers (Alliance, Rosalin, and Bintang). Data are presented as the mean of three replicates, while bars indicate the standard deviation. Means with different letters are significantly different (DMRT, *p* < 0.05).

Application of SNP markedly reduced the time to stem bending for the three different gerbera cultivars (Alliance, Rosalin, and Bintang). However, its positive effects on delaying stem bending were dose- and cultivar-dependent: 20 mg L^-1^ was observed to be the optimal concentration for Alliance, while 10 and 5 mg L^-1^ were the best for Rosalin and Bintang, respectively (**Figure 2**). In all cultivars, low concentrations did not sufficiently delay stem bending, which was also found for concentrations higher than the optimal levels. Sometimes, the higher concentrations hastened stem bending, even faster than controls, particularly in Rosalin.

**FIGURE 3:**
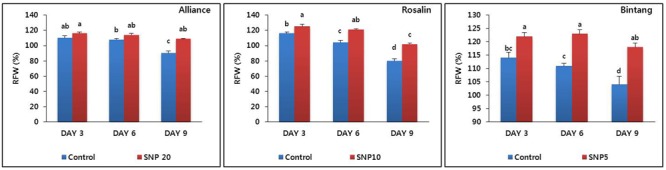
The effect of SNP on relative fresh weight (RFW) of the different gerbera cut flowers (Alliance, Rosalin, and Bintang) during the vase period (days 3, 6, and 9). Data are presented as the means of three replicates, while bars indicate the standard deviation. Means with different letters are significantly different (DMRT, *p* < 0.05).

Based on the results of vase life, we selected the optimal concentration for each cultivar (20, 10, and 5 mg L^-1^ for Alliance, Rosalin, and Bintang, respectively) and evaluated their RFWs compared to controls during the vase life period (days 3, 6, and 9). Generally, the RFWs with the treatments were higher than those under control conditions; however, the specific responses varied among the cultivars. For example, the RFWs between the control and the treatments were significantly different on day 9 for Alliance, while significant differences were observed starting from day 3 in Rosalin and Bintang (**Figure 3**). In addition, the decline in RFW throughout the vase life for the treatments was distinctly slower than for the controls in all cultivars. The RFWs of the treatments did not distinctly decline until day 6 in Alliance and Rosalin and until day 9 for Bintang. Here, the reduction in time to stem bending with the treatment concentration was strongly associated with the RFW of the cut flowers. In Alliance, the RFW of the 20 mg L^-1^ treatment was 19% higher than that of the control at day 9, when the vase life of the control ended. Similarly, in Rosalin or Bintang as well, the RFWs obtained for the respective optimal concentration (10 or 5 mg L^-1^) were also 22 or 21% higher than those obtained for the controls on day 9 or day 12, respectively, when the vase life of controls ended.

### Determination of Bacterial Density in the Vase Solutions

**FIGURE 4:**
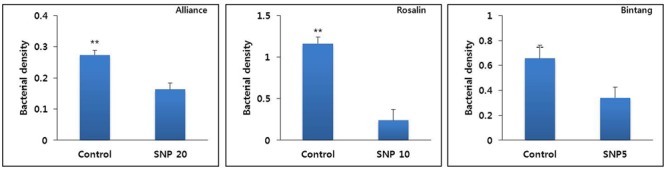
The effect of SNP on inhibition of bacterial growth in vase solutions of different gerbera cultivars (Alliance, Rosalin, and Bintang) on day 9 after treatment. Data are presented as the mean of three replicates, while bars indicate the standard deviation. Means with asterisks (^∗∗^) are significantly different (*t*-test, *p* < 0.05 or *p* < 0.01).

As shown in **Figure 4**, SNP inhibited bacterial growth in vase solutions. The bacterial densities in the vase solution of the controls were significantly higher than those in the SNP treatment solutions (**Figure 4**). The presence of a higher density in the controls than in the SNP treatments was associated with a faster rate of stem bending in controls than in the SNP treatments for all cultivars.

### Expression Level of *CCoAMOT3* and *PAL* Genes

**FIGURE 5:**
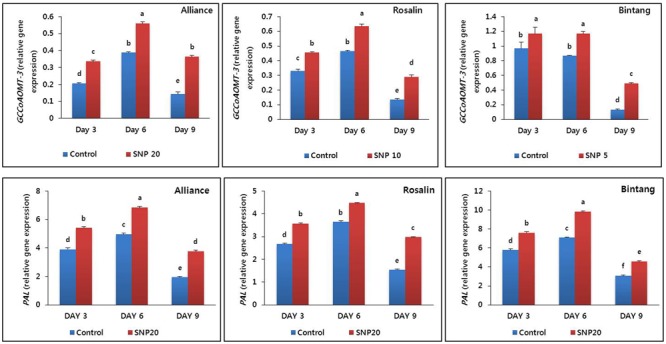
The effect of SNP on transcript levels of a lignin biosynthesis gene (*GCCoAOMT-3*) and *PAL* gene in stem segments of the gerbera cultivars (Alliance, Rosalin, and Bintang) during the vase period (days 3, 6, and 9). Data are presented as the mean of three replicates, while bars indicate the standard deviation. Means with different letters are significantly different (DMRT test, *p* < 0.05).

The lignin biosynthesis gene *CCoAMOT3* and the lignin precursor gene *PAL* were differentially expressed in stem segments (10–15 cm below flowers) during the vase life period of all cultivars. Expression levels in the SNP treatments were significantly higher than those of the controls (**Figure 5**). The expression levels were slightly different among the cultivars and the highest expression levels were observed on day 6 for both *CCoAMOT3* and *PAL*. A distinct decline in gene expression was observed on day 9, particularly in the controls.

### Antioxidant Activities

**FIGURE 6:**
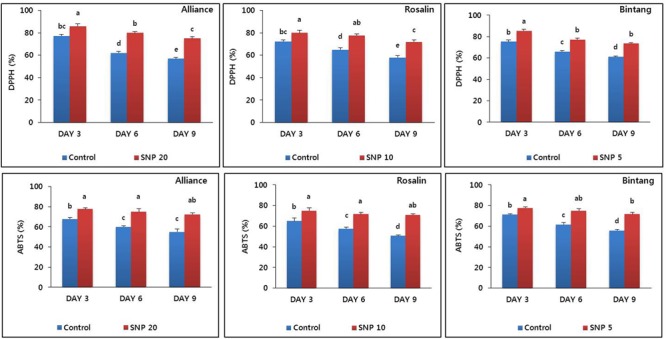
The effect of SNP on DPPH and ABTS activities in the stem segments of the gerbera cultivars (Alliance, Rosalin, and Bintang) during the vase period (days 3, 6, and 9). Data are presented as the mean of three replicates, while bars indicate the standard deviation. Means with different letters are significantly different (DMRT, *p* < 0.05).

We detected antioxidant activities, by measuring DPPH and ABTS in stem segments (about 10–15 cm below the flower where stem bending normally occurred). These molecules scavenge reactive oxygen species, which can weaken the stem strength of cut flowers by damaging cell membranes. In all cultivars, the activities obtained by SNP treatment were significantly higher than the controls across days (**Figure 6**). In Alliance, the amounts of DPPH and ABTS under control conditions during the vase life declined from 77.33 and 68% at days 3 to 57 and 55% to day 9, respectively, while these declines under control conditions are much less significant, the DPPH and ABTS remains at very high levels in SNP-treated plants although their amounts are also reduced from 86 and 77.66%, to 75 and 72.3%, respectively. A similar trend was also observed in Rosalin and Bintang.

### Scanning Electron Microscopy

**FIGURE 7:**
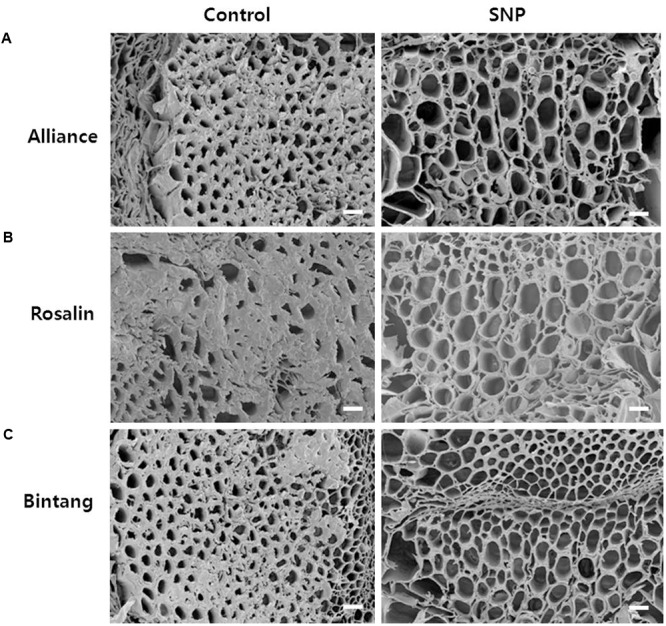
Comparison of cut flower stem surfaces in controls and SNP treatments of **(A)** Alliance, **(B)** Rosalin, and **(C)** Bintang on day 9 after treatment (Scale bars indicate 200 μm).

**FIGURE 8:**
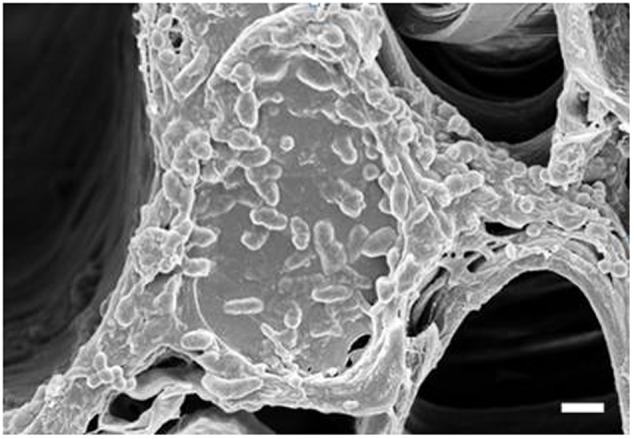
Observation of bacteria on the xylem surface of control stems of ‘Rosalin’ on day 9 after treatment (Scale bar indicates 20 μm).

To determine if the xylem status of flower stems was associated with vase life, we investigated the xylem status of the flower stems during the vase life of all cultivars using scanning electron microscopy. The xylem statuses observed on days 0 and 3 were similar and clear in both controls and treatments (data not shown). However, blockage of xylem was found in controls on day 9, when their RFWs started to decline, but little xylem blockage was detected in any of the treatments in comparison with the controls (**Figure 7**). By day 9, xylem, blockages had formed in all cut stem surfaces due to the growth of bacteria (**Figure 8**). Their growth was more serious in the controls of Rosalin than in the controls of Alliance and Bintang. The xylem blockages observed negatively affected water transport and caused earlier stem bending than in the treatments.

## Discussion

The vase life of cut gerbera flowers is ended when cut stems exhibit bending or breaking. Although the mechanism that causes stem bending remains unclear, previous studies have demonstrated that several factors, such as turgor loss, ethylene production, xylem blockage, PAL activity, lignin content, and genetic background, are associated with the mechanism that causes stem bending (van Doorn and de Witte, 1994; Werett et al., 1996; Ferrante et al., 2007; Li et al., 2009). Recently, SNP, a nitric oxide donor known to be a signal molecule involved in biotic and abiotic stress tolerance, has been increasingly used to extend the vase life of cut flowers, such as rose, gladiolus, and carnation (Zeng et al., 2011; Liao et al., 2013; Dwivedi et al., 2016). Their vase lives were extended by SNP inhibiting ethylene production and promoting antioxidant activity. In addition, SNP inhibited the growth of bacteria, such as *Clostridium sporogenes, Staphylococcus aureus*, and *Escherichia coli*, which are the most commonly observed species in the xylem of cut flowers (Joannou et al., 1998; Seabra et al., 2010; Martínez-Ruiz et al., 2011; Cardozo et al., 2014). It also positively affected lignification (Böhm et al., 2010). However, whether SNP participates in the mechanisms causing stem bending of cut gerbera flowers has not been yet investigated. Therefore, in this study, we investigated the role of SNP in the mechanisms causing stem bending.

In this study, SNP promoted the vase life of the cut flowers via a delay in the time to stem bending; however, the effects were found to be dose- and cultivar-dependent. In Alliance, all concentrations could extend vase life compared to the controls and 20 mg L^-1^ was the best among the treatments to improve vase life. In contrast, moderate concentrations (10 and 5 mg L^-1^) were the best for the vase life of Rosalin and Bintang, respectively, and lower (1 mg L^-1^) or higher (15 and 20 mg L^-1^) concentrations did not promote vase life in those cultivars; in fact, the higher concentrations shortened vase life compared to the control in Rosalin. Similar findings have been observed in other cut flowers (phlox, rose, gladiolus, and carnation), in which SNP at lower concentrations was less effective to promote vase life, while high concentrations shortened vase life compared to the controls (Sankhla et al., 2003; Zeng et al., 2011; Liao et al., 2013; Dwivedi et al., 2016). In the present study, it was observed that the SNP dose that was best for one cultivar was not suitable for another; thus, variation in the optimal dose of SNP among cultivars for the enhancement of their vase life could result from differences in their genetic background. In addition, among the cultivars, the controls of Bintang exhibited delayed stem bending compared to that of the other cultivars, and the variation observed among the cultivars was consistent with the results of a previous study by Ferrante et al. (2007). Moreover, stem bending associated with genetic background has been reported in previous studies (De Jong and Garretsen, 1985; Werett et al., 1996). Thus, the choice of cultivars that can withstand stem bending is likely a good strategy to extend vase life. Therefore, parameters (such as RFW, bacterial density, expression of lignin biosynthesis gene, antioxidant activities, and xylem blockage) that are associated with stem bending and longevity of cut flowers were further investigated between the control and the optimum concentration for each cultivar.

Relative fresh weight was found to be positively associated with the stem turgidity of cut flowers. The RFWs obtained for the optimal concentrations were significantly higher than the controls in all cultivars, especially on day 9 for Alliance. In fact, petal wilting and senescence (water loss) did not occur in the flowers; however, shrinkage of the stem (water loss) was distinctly noticed on day 7, especially in the stem 10–15 cm below the flower where bending occurred on day 9. Therefore, it is suggested that the difference in RFWs between the controls and treatments was due to stem shrinkage and bending and SNP can delay stem bending by enhancing stem turgidity.

In addition, stem bending was associated with bacterial density in the vase solution. The densities measured in the controls were significantly higher than those in the SNP treatments on day 9. van Doorn and de Witte (1994) and Perik et al. (2014) also claimed that a high level of bacteria caused rapid stem bending in gerbera cultivars. Thus, it was obvious that inclusion of SNP inhibited bacterial growth and delayed stem bending. An association of delayed stem bending and low bacterial concentration has been reported (Perik et al., 2014). In addition, this finding supports the results of previous reports describing the bactericidal effects of SNP, which is most commonly observed in the xylem of cut flowers (Joannou et al., 1998; Seabra et al., 2010; Martínez-Ruiz et al., 2011; Cardozo et al., 2014). Many researchers reported that sliver nitrate and sliver nanoparticles, which are primarily known as ethylene inhibitors and antimicrobial agents, delayed stem bending of gerbera flowers (Nair et al., 2003; Meman and Dabhi, 2006; Prashanth and Chandrasekhar, 2007; Liu et al., 2009; Solgi et al., 2009). These authors stated that strong inhibition of bacterial counts in vase water and stem vessels, in which high bacterial counts can result in bacterial xylem blockage, were associated with the delay in stem bending, whereas the role of ethylene in stem bending was not discussed. Therefore, delayed stem bending was likely caused by inhibition of bacterial growth because gerbera is not sensitive to ethylene.

Previous studies have demonstrated that lignification is also positively associated with stem strength (Pilate et al., 2004; Do et al., 2007; Ferrante and Serra, 2009; Li et al., 2009; Ma, 2009). In the present study, a lignin biosynthesis gene (*GCCoAOMT-3*) and a lignin precursor gene, *PAL*, were highly expressed when treated with SNP, compared to controls, in stem segments about 10–15 cm below the flower where bending occurred. Thus, the delay in stem bending induced by SNP may be due to its stimulation of lignin biosynthesis, which can enhance mechanical strength and increase the hydrophobicity of xylem vessels (Pilate et al., 2004; Do et al., 2007). The role of SNP in lignification has also been reported in soybean seedlings (Böhm et al., 2010); however, in the present study, SNP increased lignification by the strong induction of *GCCoAOMT-3* and *PAL* in stem segments. The roles of PAL activity that enhance lignification and delay stem bending have previously been reported for gerbera flowers (Brown, 1990; Mencarelli et al., 1995; Ferrante et al., 2007; Ferrante and Serra, 2009). In addition, PAL’s role in the induction of phenol accumulation that leads to tissue lignification, has been observed in several plants (Iriti and Faoro, 2003; Liu et al., 2005). In addition, the association of lignification and *CCoAOMT* gene expression has been previously reported in several plants (Ye, 1997; Zhao et al., 2004; Wang et al., 2013). Here, it is suggested that SNP may enhance lignification in stem segments by induction of the detected genes and that higher lignification might contribute to stem rigidity.

Higher antioxidant activities can scavenge more reactive oxygen species, which can damage plant cell membranes and cause plant deterioration. The association of high antioxidant activities and flower longevity has been reported in several cut flowers (Chakrabarty et al., 2007; Ezhilmathi et al., 2007; Kumar et al., 2008). Thus, in the present study, we measured antioxidant (DPPH and ABTS) activities contained in stems treated with SNP and the control. Here, higher antioxidant activities were detected in stems treated with SNP than in stems of the controls for all cultivars, consistent with the delay in stem bending. Therefore, we suggest that SNP is involved in stem bending by maintaining antioxidant activity during vase life. However, during the vase life period, differences in the maintenance of antioxidants among the cultivars could have resulted from differences in genotype and the interaction between SNP dose and each cultivar. The role of SNPs in extending the vase life of some cut flowers by maintaining antioxidant activities has also been reported (Zeng et al., 2011; Dwivedi et al., 2016).

Rapid stem bending of cut gerbera caused by xylem blockage has been reported (Liu et al., 2009). Hence, we investigated xylem status in controls and SNP treatments during vase life. Expectedly, xylem status was associated with stem strength. Xylem status, as observed in controls and SNP treatments, was similar on days 3 and 6; however, on day 9, when control stems of Alliance and Rosalin bent, xylem blockage was more extensive in the controls than in the SNP treatments. Xylem blockage in the control stems of Alliance and Rosalin was more extensive than that in Bintang, whose stems bent on day 12. Xylem blockage at the stem surface, due to bacterial growth, has been reported in previous studies (van Doorn, 1997; Ranwala and Miller, 2009). In the present study, less xylem blockage in the SNP treatments would be expected to be due to SNP’s antibacterial activity, which has been previously reported (Joannou et al., 1998; Martínez-Ruiz et al., 2011; Cardozo et al., 2014).

Taken together, stem bending in the gerbera cultivars is likely to be associated with many parameters, particularly lignification, antioxidant activities, PAL activity, bacterial density, and xylem blockage. In addition, the choice of a proper cultivar that can delay or eliminate stem bending is a good approach to extend vase life. In this study, the inclusion of SNP in vase solutions for 24 h enhanced expression of a lignin biosynthesis gene and *PAL* gene, maintained antioxidant activities, reduced bacterial density, and reduced xylem blockage in stem segments, leading to a delay in stem bending. These results suggest that SNP could delay stem bending of gerbera cultivars by mechanisms increasing the mechanical strength of cut stems.

## Author Contributions

AN and KL designed the study, conducted the experiments, and wrote the manuscript. TA and KK assisted the experimentation and data analysis. CK supervised experiments at all stages and performed critical revisions of the manuscript. All authors read and approved the final manuscript.

## Conflict of Interest Statement

The authors declare that the research was conducted in the absence of any commercial or financial relationships that could be construed as a potential conflict of interest.
